# A near-complete genome assembly of *Fragaria iinumae*

**DOI:** 10.1186/s12864-025-11440-0

**Published:** 2025-03-14

**Authors:** Haiyuan Du, Yiying He, Maoxian Chen, Xu Zheng, Daping Gui, Jixing Tang, Yu Fang, Yiwei Huang, Hong Wan, Jiwei Ruan, Xin Jin, Andan Zhu

**Affiliations:** 1 Germplasm Bank of Wild Species & Yunnan Key Laboratory of Crop Wild Relatives Omics, Kunming Institute of Botany, Chinese Academy of Sciences, No. 132 Lanhei Rd, Heilongtan, Kunming, 650201 Yunnan China; 2 University of Chinese Academy of Sciences, Beijing, 100049 China; 3 Horticultural Research Institute, Yunnan Academy of Agricultural Sciences, Kunming, 650205 Yunnan China; 4 Flower Research Institute, Yunnan Academy of Agricultural Sciences, Kunming, 650205 Yunnan China

**Keywords:** *Fragaria iinumae*, Near-complete genome, Telomere repeat contraction/expansion, Early branching

## Abstract

*Fragaria iinumae*, a diploid progenitor species of octoploid strawberries, likely occupies a basal position within the genus *Fragaria*. In this study, we report a near-complete genome assembly of *F. iinumae* v2.0, totaling 241.14 Mb with a contig N50 of 33.31 Mb. We identified 14 telomeric and 7 centromeric regions across its seven chromosomes. Compared to previous assemblies, *F. iinumae* v2.0 demonstrates substantial improvements in both genome continuity (gaps reduced from 29 to 0) and annotation completeness, including the annotation of 4,144 new genes and 395 new gene clusters. Notably, several large structural variants were identified between the *F. iinumae* v1.0 and *F. iinumae* v2.0 genomes, with most gaps in the v1.0 assembly overlapping with structural variant breakpoints. Additionally, we found a significant expansion of telomeric repeats in the B subgenome of octoploid strawberries compared to *F. iinumae*. Interestingly, two telomeres consistently exhibited low repeat abundance in both the diploid and octoploid B subgenomes, suggesting significant contraction early in the evolution of *F. iinumae*. Furthermore, through multiple lines of genomic evidence—including phylogenetic analyses, genetic distance matrices, a burst of LTR insertions, and the distribution of NLR resistance genes, we conclude that *F. iinumae* may represent an early-diverging lineage within the strawberry genus. This updated assembly provides a crucial genomic resource for understanding of the origin and structural dynamics of the strawberry genus and facilitates further exploration of genome-wide consequences of polyploidy.

**Supplementary Information:**

The online version contains supplementary material available at 10.1186/s12864-025-11440-0.

## Background

The strawberry genus, *Fragaria*, is characterized by significant genomic diversity resulting from interspecific hybridization, polyploidy and the diversification of domesticated trait diversity, making it a particularly valuable genetic system for studying polyploidy and domestication [1, 2]. It comprises approximately 25 recognized wild species spanning six ploidy levels. It also includes the cultivated strawberry, *Fragaria* × *ananassa* (2n = 8x = 56), which was domesticated through interspecific hybridization between the two wild octoploid progenitors, *Fragaria chiloensis* and *Fragaria virginiana* [1, 2]*.* Recent studies suggest that allo-octoploid strawberries originated from a biological reunion between a diploid A-genome species closely related to *Fragaria vesca* and a possibly extinct hexaploid species with three sets of similar subgenomes (B and/or B-like) [3–8]. The closest living relative of the progenitor of these subgenomes is *Fragaria iinumae* [3], a species primarily distributed in the alpine mountains of Japan and eastern Russia. *F. iinumae* exhibits distinct morphological features among diploid strawberries, such as deciduous leaves, 6–9 petals, and spinuliferous pollen grains [1, 9, 10]. Molecular phylogenetic studies indicate that *F. iinumae* likely occupies a basal position in the phylogeny, making it crucial for understanding the origin of multiple polyploidization events in *Fragaria* [5, 11].

Beyond the inference of polyploid parentage in strawberries, recent studies have increasingly focused on the genome-wide consequences of polyploidy, particularly with the availability of high-quality octoploid genomes and functional data [7, 12, 13]. For instance, the merger of genomes from different progenitors in octoploid strawberry has led to homoeologous exchanges, particularly from the A to B subgenomes, as well as homoeolog expression bias [7, 12]. Additionally, shifts of cytoplasmic content during polyploidization events have been documented, with octoploid strawberries possessing A-derived (from *F. vesca*) chloroplast genomes and B-derived (from *F. iinumae*) mitochondrial genomes [14, 15]. To facilitate comparative genomic analyses, high-quality genomes of descendants of ancestral species are essential. However, a significant gap remains in our understanding of the genome structure of *F. iinumae*.

To date, two versions of the *F. iinumae* genomes have been published, an initial genome draft by Hirakawa et al. (2014) [16] using second-generation sequencing, and a chromosome-scale assembly by Edger et al. (2020) [17], constructed using third-generation PacBio and next-generation Illumina data. While the chromosome-scale assembly (hereafter referred to as *F. iinumae* v1.0) represents a significant improvement, it contains at least 29 gaps across the seven chromosomes, limiting its utility for comparative genomics studies. Here, we report a high-quality, T2T chromosome-level assembly of *F. iinumae*, identify a significant expansion of telomere repeats in the B subgenome of octoploid strawberry compared to *F. iinumae*, and confirm the phylogenetic position of *F. iinumae* relative to strawberry diploid species.

## Results and discussion

### Assembly and Annotation of the *F. iinumae* genome

**Fig. 1:**
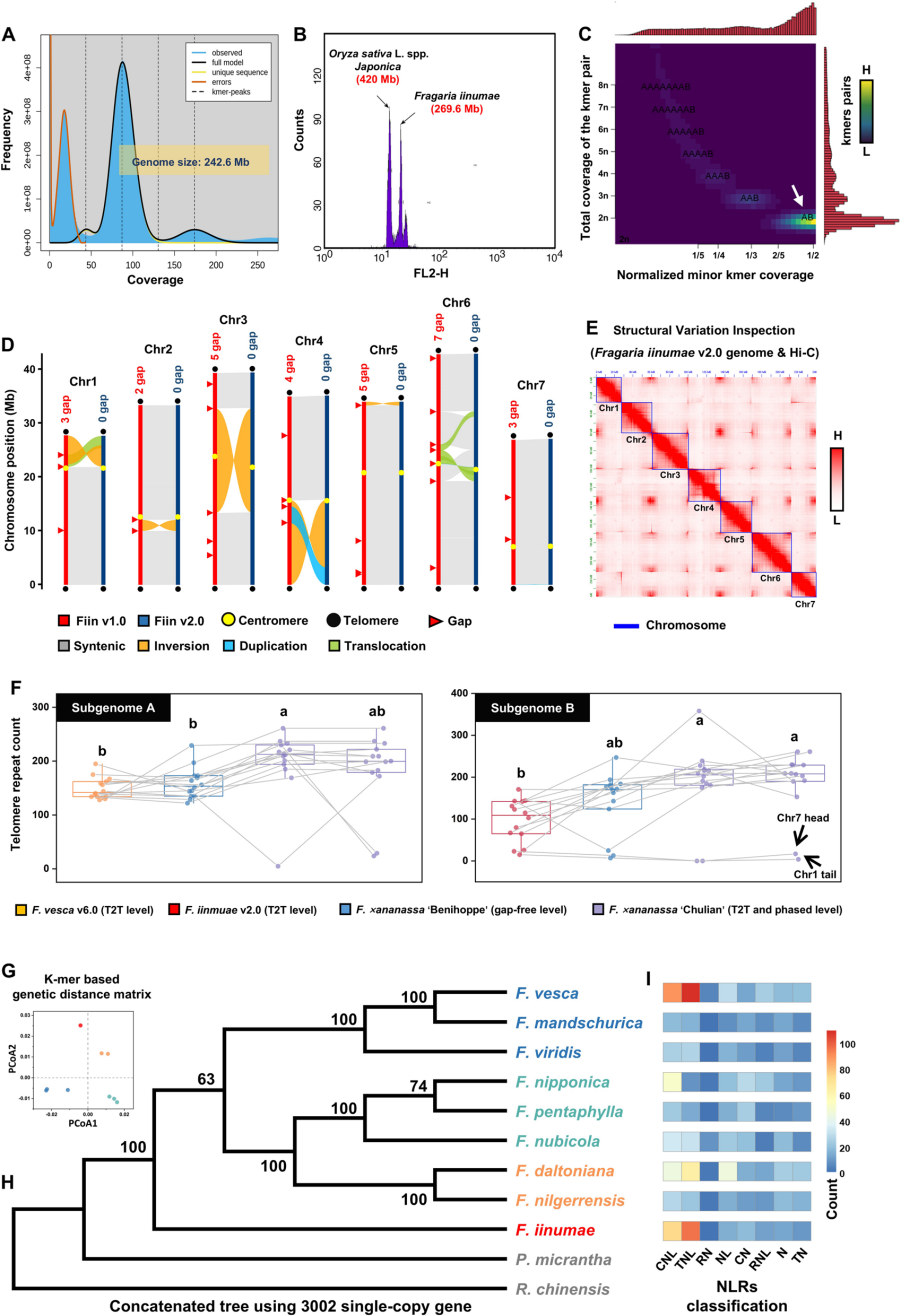
Genome assembly and phylogenomic analyses of *Fragaria iinumae*. **A** Genome size estimate for *F. iinumae* based on 21-mer k-mer analysis. **B** Genome size determination using flow cytometry with *Oryza sativa* L. ‘Japonica’ as an internal reference. **C** Smudge plot analysis indicating the ploidy level of *F. iinumae*. **D** Schematic representation of chromosomal features in *F. iinumae* v1.0 and v2.0, including telomeres, centromere, gaps, and collinear blocks. **E** Hi-C interaction heatmap of *F. iinumae* v2.0. **F** Comparison of telomere repeat abundance among *F. vesca* and subgenome A (one-way ANOVA with Duncan’s multiple range test). Comparison of telomere repeat abundance among *F. iinumae* and subgenome B (one-way ANOVA with Duncan’s multiple range test). **G** Principal Coordinate Analysis (PCoA) based on the genetic distance matrix of nine diploid strawberry genomes. **H** Phylogenetic tree constructed from nine diploid *Fragaria* species. **I** Number of NLRs identified across the nine diploid *Fragaria* species

The *F. iinumae* v2.0 genome (2n = 2x = 14) was assembled using 26.7 Gb PacBio continuous long reads (CLR), resulting in a total of 17 contigs with an assembly size of 241.14 Mb and a contig N50 of 33.31 Mb (Table S1). All contigs were anchored and oriented with 33.36 Gb of Hi-C data, yielding seven super-scaffolds ranging from 27.04 Mb to 42.95 Mb (totaling 239.57 Mb sequences) with three gaps. The assembly size closely aligns with the estimate derived from k-mer spectrum analysis (242.6 Mb) (Fig. 1A-C; Table S1). The genome size measured by flow cytometry differs by approximately 10% (Fig. 1A-C; Table S1), likely reflecting uncertainties in the exact reference genome size and potential biases in measurement methods [18, 19]. Three small scaffolds (61.9–1442 Kb), primarily derived from highly abundant repetitive sequences and mitochondrial (MW537839.1) genomes [15], were excluded from further analyses (Fig. S1). Gaps in the seven chromosomes were successfully filled using a combination of PacBio long reads and Illumina short reads, resulting in a gap-free genome assembly for *F. iinumae* (v2.0). In contrast, the previous *F. iinumae* v1.0 assembly contained at least 29 gaps across seven chromosomes (Fig. 1D; Table S1).

The predicted repetitive elements in the *F. iinumae* genome v2.0 account for 44.56% of the total genome size, and a total of 28,198 gene models were annotated (Table S1; Table S2). This includes 4,144 additional genes and 395 new annotated gene clusters compared to the 23,665 genes annotated in the *F. iinumae* v1.0 genome (Table S1; Fig. S3A-D).

#### Quality assessment of the *F. iinumae* genome v2.0

Several metrics were employed to validate the assembly and annotation quality of the *F. iinumae* v2.0 genome (Table S1). The overall coverage of PacBio reads across the genome is consistent (Fig. S2). In terms of completeness, the LTR assembly index (LAI) of 16.3 indicates a high level of completeness in the LTR-rich regions, placing the assembly within the reference-quality range (Table S1). Additionally, telomeric satellites were detected at the ends of all seven pseudochromosomes (Fig. 1D; Table S1), and centromeric satellite motifs were detected (Fig. 1D; Table S3). Moreover, over 99% of k-mers from the PacBio reads were detected in the *F. iinumae* v2.0 genome (Table S1). The k-mer-based quality value (QV) of 47.6 demonstrates high accuracy at the single-base level for the genome (Table S1). In terms of annotation completeness, 96.4% of BUSCO conserved orthologs and 96.2% of conserved HOGs were identified (Figure S3A-D; Table S1). These values slightly surpass those of the *F. iinumae* v1.0 genome, which identified 95.4% of BUSCOs and 96.1% of HOGs (Table S1). Collectively, these results indicate that the *F. iinumae* v2.0 genome represents a near-complete genome assembly with high complement in gene annotation.

Notably, several large structural variations (> 1 Mb) were identified between the *F. iinumae* v1.0 and v2.0 genomes. A particularly large inversion (> 20 Mb) was observed in chromosome 3. However, this inversion was only detected between the *F. iinumae* v1.0 genome and other *Fragaria* species [5], but not in the *F. iinumae* v2.0 genome (Fig. S4). The accuracy of the *F. iinumae* v2.0 assembly was further confirmed using Hi-C reads (Fig. 1E; Figure S5A), providing strong evidence for the integrity of the v2.0 assembly. The 29 gaps in the *F. iinumae* v1.0 assembly were found to coincide with structural variant breakpoints. To further assess the structural variations (SVs), we randomly selected 15 large SVs and examined the continuity of reads within a 10 kb region upstream and downstream of these breakpoints. The results revealed that 14 breakpoints in the v1.0 genome exhibited gaps in the CLR reads, whereas the CLR reads in the v2.0 genome showed high continuity at these locations (Fig. S6), suggesting that some structural variants identified between the v1.0 and v2.0 assemblies may be false-positives (Fig. 1E; Figure S5B). Interestingly, while seven centromeric regions were found in both v.10 and v2.0, a more complete centromere structure of Chr4 was observed in v2.0 compared to v1.0 (Fig. S7). These findings highlight the utility of Hi-C data and advancements in assembly algorithms for constructing high-quality genomes and facilitating comparative genomics.

### Variations in telomere repeat abundance between *F. iinumae* and the B subgenomes of octoploid strawberries

The T2T chromosome-level *F. iinumae* v2.0 genome provides a unique opportunity to explore variations in telomere repeat abundance (Fig. S5C). The diploid references *F. vesca* v6.0 [20], *F. iinumae* v2.0 (this study), and the cultivated octoploid strawberries *F.* × *ananassa* ‘Benihoppe’ [21] and *F.* × *ananassa* ‘Chulian’ (including both haplotypes) [22] were selected to compare telomere repeat abundance between diploid and octoploids. We found that the *F. vesca* genome and the A subgenome of ‘Benihoppe’, as well as the *F. iinumae* genome and the B subgenome of ‘Benihoppe’, exhibited closely matched chromosome lengths (two-sided test: *P* < 0.001; R^2^ > 0.9) and a high degree of genome-wide colinearity (Figure S8A-C). Interestingly, when comparing the telomere repeat abundance in *F. vesca* to that of the A subgenome in octoploids, we observed an expansion trend, though this difference was not statistically significant (two-sided test: *P* > 0.05). In contrast, when comparing the telomere repeat abundance in *F. iinumae* to that of the B subgenome in octoploids, we found a significant expansion (two-sided test: *P* < 0.05) (Fig. 1F).

Furthermore, we found that LTR-retrotransposon abundance in the B subgenome was significantly higher (two-sided test: *P* = 1.42E-106) than in the A subgenome (Fig. S8D), which may contribute to the notable expansion of the (sub-)telomeric regions in the B subgenome, potentially stabilizing the genome. Interestingly, the telomeric repeats of Chr1_tail and Chr7_head regions were found in low abundance (0–23 counts) in both *F. iinumae* and the B subgenome of all tested octoploids (Fig. 1F). This suggests that these two telomeres underwent significant historical contraction early in the evolution of *F. iinumae*, and that these low-abundance telomeric repeats were retained in octoploids during hybridization and polyploidization. The contraction may be attributed to recombination-mediated deletion events, or alterations in telomere-associated protein activity. These mechanisms could reflect a unique adaptive response or genomic constraint in *F. iinumae*, warranting further investigation to fully understand its biological significance during polyploidization.

### *F. iinumae* may represent one basal clade in the diploid strawberry lineage

Previous studies have provided differing interpretations of the phylogenetic position of *F. iinumae*. To resolve the branching order of diploid strawberries and accurately place *F. iinumae*, we used the high-quality *F. iinumae* v2.0 genome, along with *Rosa chinensis* [23] and *Potentilla micrantha* [24] as outgroups, to construct a phylogenetic tree among nine diploid wild strawberry species [5, 7, 20, 23–25]. A set of 3,002 single-copy ortholog sets from these 11 species were used for sequence alignment and tree construction, confirming that *F. iinumae* may represents an early divergence within the strawberry genus, being sister to the remaining diploids (Fig. 1H; Figure S9A). The finding was further supported by a genome-wide nuclear and plastid genome genetic distance matrix (Fig. 1G; Fig. S10).

We also identified 3139, 1187, and 2771 lineage-specific LTR retrotransposons in representative species from each clade (*F. iinumae*, *F. vesca*, and *F. nipponica*), suggesting that the earliest specific LTR insertion event occurred in *F. iinumae* (Figure S9B). Early divergent species, such as *F. iinumae*, which have undergoing undergone complex environmental adaptations over a prolonged evolutionary period [26], likely accumulate a large repertoire of disease resistance genes to cope with diverse pathogens. Thus, we identified and classified the number of NLRs in the nine diploid species, revealing that *F. iinumae* harbored the highest number of the CNL and TNL NLRs (mainly involved in pathogen recognition) among the species examined (Fig. 1I). The exclusion of *F. vesca* may be attributed to its history of widespread cultivation, during which a large number of resistance genes were artificially introduced (Fig. 1I).

Taken together, these results suggest that the *F. iinumae* is one early diverging lineage among all extant wild diploid strawberries and provides a genomic foundation for the study of octoploid strawberries.

## Conclusions

In summary, our chromosome-level genome assembly of *F. iinumae* v2.0 is nearly complete, with only a few small non-plasmid repetitive fragments remaining unanchored. This high-quality assembly, along with comprehensive gene annotations, provides a robust reference for genomics analysis. Compared to *F. iinumae*, subgenome B exhibits a significant expansion in telomere abundance, while the low abundance of telomeric repeats at Chr1_tail and Chr7_head suggests a historical contraction in *F. iinumae*. This telomere contraction may stem from recombination-mediated deletions, potentially linked to genome stability during evolution. This reference genome also supports the hypothesis that *F. iinumae* represents one of the earliest diverging lineages within the diploid strawberry group, suggesting that *F. iinumae* may be a critical diploid ancestor in the evolution of octoploid strawberries. Its basal phylogenetic position suggests the retention of ancestral genomic features, offering valuable insights into polyploid formation and adaptation in *Fragaria*. However, a single *F. iinumae* genome may not capture the full genetic diversity of this species. Therefore, future studies incorporating broader sampling are required, and phylogenetic reconstructions based on pan-genome data will be essential to solidify this conclusion.

## Methods

### Plant materials

Plants of *Fragaria iinumae* were grown in the greenhouse (temperature: 15–25 °C; humidity: 30–60%; light: natural) of Kunming Institute of Botany (Kunming, China). Fresh young leaves were collected from a single plant to minimize sequencing and assembly errors caused by inter-individual variation. The sampled leaves were immediately frozen in liquid nitrogen for subsequent use.

### PacBio library preparation and sequencing

Total genomic DNA was extracted from the leaves using the Plant Genomic DNA Extraction Kit (R30171; Shanghai Yuanye Bio-Technology Co., Ltd), following the manufacture’s protocol. RNase A was used to remove RNA contaminants, then DNA integrity was assessed via agarose gel electrophoresis (Fig. S11). For PacBio sequencing, a SMRTbell library was constructed using the SMRTbell Template Prep Kit v2.0, following the established manufacture’s protocol (Pacific Biosciences, Menlo Park, CA, USA) for plant genomes. Sequencing was performed on the Sequel II platform by Biomarker Technologies Co. (BMK), generating approximately 26.7 Gb (~ 105 ×) long-read data.

### Genome size estimation

Illumina data [15] was used to estimate genome size of the sequenced *F. iinumae* plant material. K-mer counting was performed using Jellyfish (v2.2.10) [27], and histogram data transformation was conducted using the ‘*histo*’ function with the parameter ‘-h 5000’. Genome size was estimated using GenomeScope (v2.0) [28]. Flow cytometry is also used to estimate genome size. Briefly, suspensions of each sample and the internal reference sample (*Oryza sativa* L. ‘Japonica’) were mixed, and a BD FACSCalibur flow cytometer was used to detect the stained cell nuclei in suspension samples. Ploidy level was further estimated using Smudgeplot (v0.2.3dev) [28].

### Hi-C sequencing

For Hi-C library construction, 1–4 ug of high-quality DNA was used with the Mate-pair Kit. Concentration was assessed using Qubit, and quality control was performed via Q-PCR and GX analysis. Fresh leaves were used for in vivo cross-linking with 2% formaldehyde, and the purified nuclei were digested with HindIII enzyme. The ligated DNA was then sheared and size-selected into 300–600 bp fragments for library construction. Paired-end sequencing was performed on the MGISEQ-T7 platform, generating approximately 33.4 Gb Hi-C reads (Number of reads: 223,046,478; Reads quality: 37; Average length: 150 bp; GC content: 41%) for downstream analyses.

### Genome assembly and pseudochromosome construction

Four cells of PacBio CLR data were assembled using NextDenovo (v2.5.2) [29]. The assembly was polished through three iterations with both PacBio and Illumina data using NextPolish (v1.3.1) with default parameters. The Hi-C datasets were then utilized to anchor the assembled contigs onto seven super-scaffolds, representing the seven pseudochromosomes of *Fragaria iinumae*, using 3D-DNA (v18011412). Contig sorting and orientation were conducted with Juicebox (v1.11.08) and manually adjusted manually as necessary. Whole-genome synteny between the seven *F. iinumae* pseudochromosomes and the published diploid *Fragaria vesca* genome was analyzed using MUMmer (v3.1) [30] to confirm the chromosome orientation and nomenclature.

### Gap filling

Following chromosome anchoring, three gaps remained in the *F. iinumae* genome. PBJelly (v15.8.24) [31] with parameters “-minMatch 8 -minPctIdentity 70 -bestn 1 -nCandidates 20 -maxScore -500 -nproc 32 -noSplitSubreads”, which were employed for gap filling based on PacBio reads. The output assembly was further used SoapDenovo2-GapCloser (v1.12) [32] with parameters were adjusted to max_rd_len = 149, avg_ins = 408 and asm_flags = 4, which were employed for gap filling based on Illuminareads. To prevent reintroduction of mis-joints, 3D-DNA was reapplied for contig anchoring, removing sequences lacking signals in the Hi-C heatmap. Finally, ALLHIC (v0.9.8) [33] was used to generate a genome-wide Hi-C heatmap.

### Genome evaluation

The quality of the assembly was evaluated by aligning PacBio reads to the genome using Winnowmap (v2.03) [34] with parameter ‘–secondary = no -ax map-pb’. Read alignments with a map quality of 60 were retained and used to calculate the average depth in 10 kb windows. The completeness of the assembled genome was evaluated using BUSCO (v5.2.2) [35] with the Eudicots_odb10 library. Merqury (v1.3) [36] was utilized to assess the overall base quality value (QV) and genome completeness using Illumina data.

## Genome annotation

Repetitive elements in the *Fragaria iinumae* genome were identified using RepeatModeler (v2.0.4) [37]. Repetitive sequences were predicted using RepeatMasker (v 4.1.4) [38] with default parameters and the de novo library generated by RepeatModeler. LTR_Finder (v1.07) [39] was employed for LTR identification, and LTR_retriever (v2.9.0) [40] was used to identify LTR-RTs and generate a non-redundant LTR-RT library. The LAI value was calculated using the LAI function within LTR_retriever (v2.9.0) [40, 41].

For protein-coding gene prediction, a combination of de novo, homology-based, and transcriptome-based prediction methods was utilized. De novo gene prediction was performed using Genscan (v3.1) [42], Augustus (v2.5.5) [43], GlimmerHMM (v3.0.4) [44], SNAP (v2006–07‐28) [45] and GeneID (v1.4) [46] with default parameters. Homology-based gene prediction was conducted using GeMoMa with default parameters (v1.3.1) [47] with default parameters, utilizing protein sequences from *Arabidopsis thaliana*, *Fragaria vesca*, *Malus domestica*, and *Rosa chinensis*. Transcriptome-based prediction was derived from a de novo transcriptome assembly using Trinity with default parameters (v2.8.5) [48], with further refinements using PASA (v2.0.2) with default parameters [49]. Gene predictions from these three methods were integrated into a consensus gene model using EVidenceModeler (v1.1.1) [50] with default parameters. Classification of NLR genes were performed using Resistify (v0.2.0) with default parameters [51].

### Telomere and centromere identification

Quartet (v1.2.0) [52] was employed for genome-wide telomeric identification with parameter ‘-c plant -m 0’ to ensure accurate telomeric repeat counting. Centromics (v0.3) (https://github.com/zhangrengang/Centromics), which integrates long-read sequencing and Hi-C data, was employed to pinpoint the putative centromeric regions. The centromeric positions identified by Centromics (v0.3) and TRF (v4.09) [53] were cross-referenced with TRF results to ensure consistency in the length and repetitive motifs of these regions. A 147 bp centromere specific satellite sequence to the whole genome using BLAST (v2.13.0) [54] with parameters ‘-evalue 1e-5’, and the results were combined to identify the potential centromeric regions (Table S3; Table S4). The centromere region was cut into 100 bp bins and the sequence similarity matrix was calculated using the StainedGlass (v0.6) [55].

### Comparative genomics analysis

Chromosomal collinearity analyses were performed with MUMmer (v3.1) [30] with the following three functions and parameters: (i) nucmer ‘–mum -D 5’; (ii) delta-filter ‘-m -i 80 -l 10,000’; (iii) DotPrep.py with default parameters. These analyses identified one-to-one chromosome correspondence between species (including assembly versions), aiding in chromosome naming. The collinearity map displays chromosome alignments in forward and reverse orientations, allowing evaluations of chromosome orientations in the *F. iinumae* assemblies. SyRI (v1.6) [56] was used to identify both genome-wide synteny regions and putative structural variations between the *F. iinumae* v1.0 and v2.0 assemblies. JCVI (v1.0.1) [57] was used to identify colinear blocks between *F. iinumae* and other *Fragaria* species. Comparative analysis provided insights into the conservation of specific genomic regions across *F.* species and validated those putative structural variations between the *F. iinumae* v1.0 and v2.0 assemblies.

Orthofinder (v2.5.4) [58] and JCVI (v1.0.1) [57] were employed to identify shared and specific gene clusters between *Fragaria iinumae* v1.0 and v2.0 genomes. Newly annotated genes in the v2.0 genome were visualized using Jvenn (https://jvenn.toulouse.inra.fr/app/example.html), and their potential functional annotations were inferred using Eggnog-mapper (v2.0) [59] with default parameters.

### Validation of structural variation

The structural variations were validated by aligning v1.0 and v2.0 PacBio reads to the *F. iinumae* genome using Winnowmap (v2.03) [34] with parameter ‘–secondary = no -ax map-pb’. Read alignments with a map quality of 60 were retained. We randomly selected 15 large structural variation breakpoints and examined the continuity of reads within a 10 kb region upstream and downstream of these breakpoints.

### Phylogenetic tree construction

Orthologous clusters among nine diploid *Fragaria* species and two *Rosaceae* species were identified using OrthoFinder (v2.5.4) with default parameters [58]. The species included *Fragaria nubicola* [5], *Fragaria daltoniana* [25], *Fragaria mandschurica* [25], *Fragaria nilgerrensis* [25], *Fragaria pentaphylla* [25] and *Fragaria viridis* [5], *Fragaria nipponica* [7], *Fragaria vesca* [20] and *Fragaria iinumae* (this study). *Rosa chinensis* ‘Old Blush’ [23] and *Potentilla micrantha* [24] were selected as outgroups, as they belong to closely related genera within the *Rosaceae* family [5]. These outgroups provided reference points for rooting the phylogenetic tree and established a baseline for resolving evolutionary relationships within *Fragaria*.

Single-copy orthologs were aligned using MUSLE (v3.8.31) with default parameters [60], and the resulting protein alignments were converted to coding sequence (CDS) alignments using PAL2NAL (v14.0) with default parameters [61]. Gene trees were reconstructed using IQ-TREE (v2.0.3) with parameters ‘-m MFP -bb 1000’ [62], and species trees were subsequently generated using ASTRAL (v5.7.8) with default parameters [63] from individual gene trees. Genetic distances of the plastid genomes [15] for the nine diploids were calculated using Skmer (v3.0.2) with parameters ‘-k 13, 17 and 21’, respectively [64].

### Identification and insertion time estimation of species-specific LTR-RTs

Species-specific k-mers were identified using SubPhaser (v1.1.5) [65]. Long terminal repeat retrotransposons (LTR-RTs) were detected using LTRharvest (v1.6.1) [66], LTRfinder (v1.07) [39], and TEsorter (v1.3.0) [67] with default parameters. The identified species-specific k-mers are mapped to the LTR-RT sequences, followed by Fisher’s exact test to identify species-specific LTR-RTs. Insertion times were estimated based on the Jukes-Cantor 69 model.

## Supplementary Information

Supplementary Material 1.

## Data Availability

Data availability The genome assembly and annotation files have been submitted to the Genome Database for Rosaceae (https://www.rosaceae.org/Analysis/24109543). All the raw genome sequencing data have been submitted to the National Genomics Data Center (https://ngdc.cncb.ac.cn/), and the accession number is CRA021252 (https://ngdc.cncb.ac.cn/gsa/browse/CRA021252).
