# Supplementary material for: A near-complete genome assembly of Fragaria iinumae
Source: BMC Genomics. 2025 Mar 14;26:253. doi: 10.1186/s12864-025-11440-0 (PMC11909947; doi:10.1186/s12864-025-11440-0)
Supplement: Supplementary file 1 — Supplementary Material 1. [file 12864_2025_11440_MOESM1_ESM.docx]

**Supplementary Data**

**Supplementary figures**


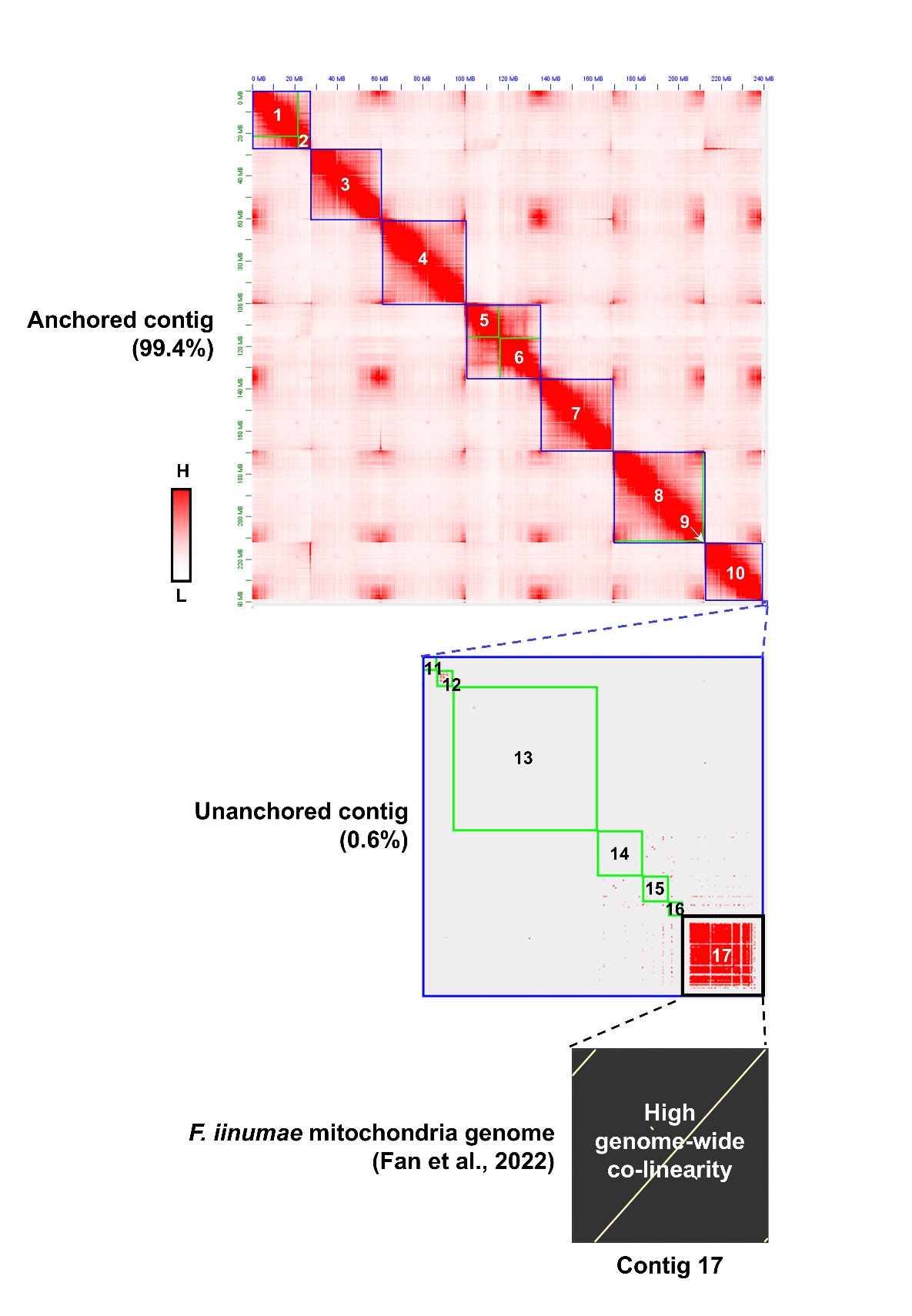


**Supplementary Figure 1 | Anchoring the *F. iinumae* v2.0 contig-level assembly to the chromosome level based on Hi-C interaction signals.** Note: A few contigs were not anchored due to the absence of Hi-C signals or because they belong to the mitochondrial genome.


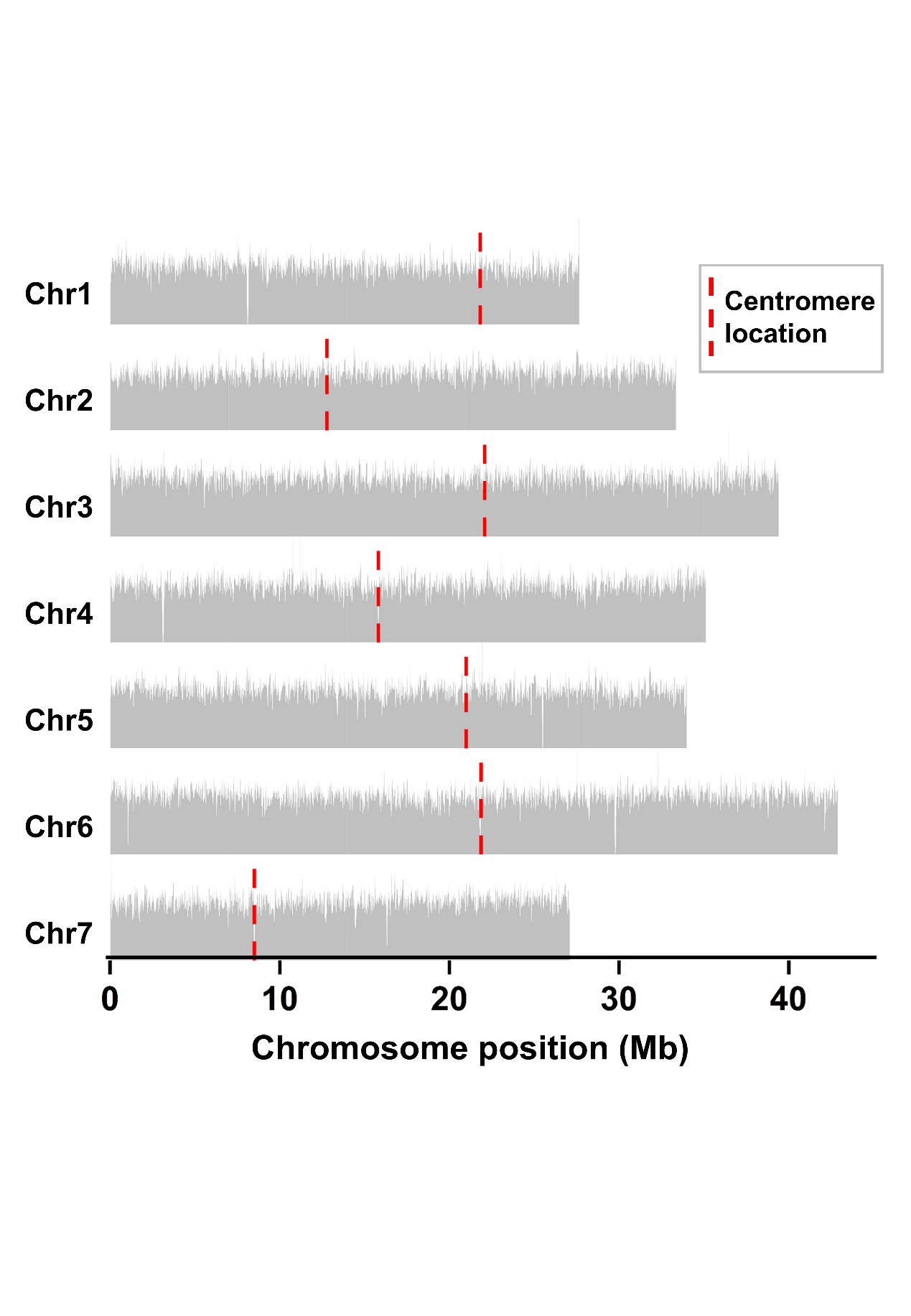


**Supplementary Figure 2 | Average depth of PacBio reads on the *F. iinmuae* v2.0 genome.** Note: Bin size: 10 kb; Map quality: 60; The red dotted line represents the location of the centromeres.


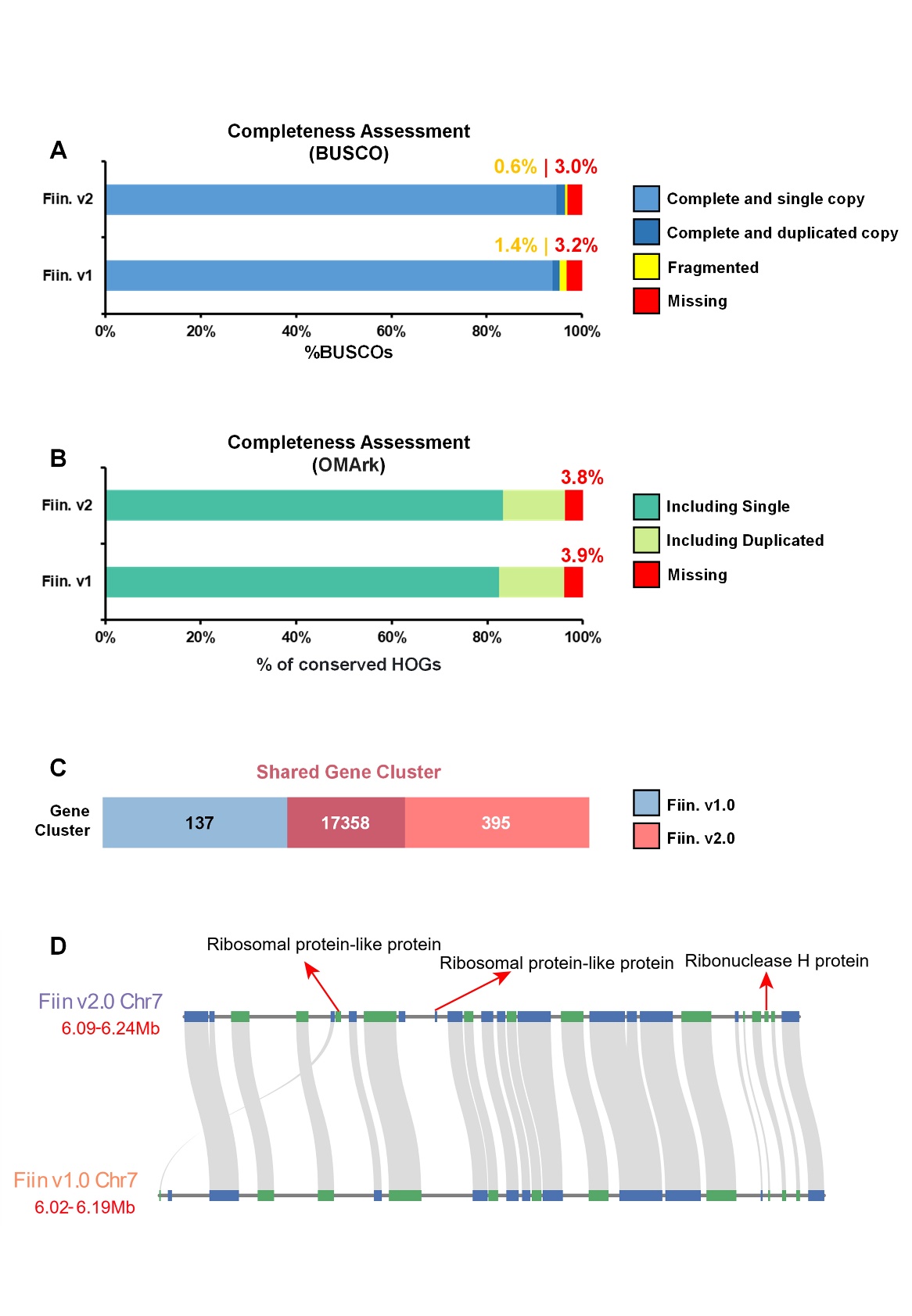


**Supplementary Figure 3 | Quality evaluation and validation of *Fragaria iinumae* genome assembly and annotation. (A)** BUSCO analysis assessing annotation completeness in *F. iinumae* v1.0 and v2.0. **(B)** Quality assessment of gene repertoire annotations in *F. iinumae* v1.0 and v2.0 using OMArk. **(C)** Number and proportion of shared and unique gene clusters between v1.0 and v2.0 assemblies. **(D)** Local gene collinearity on the short arm of chromosome 7 between v2.0 and v1.0 assemblies.


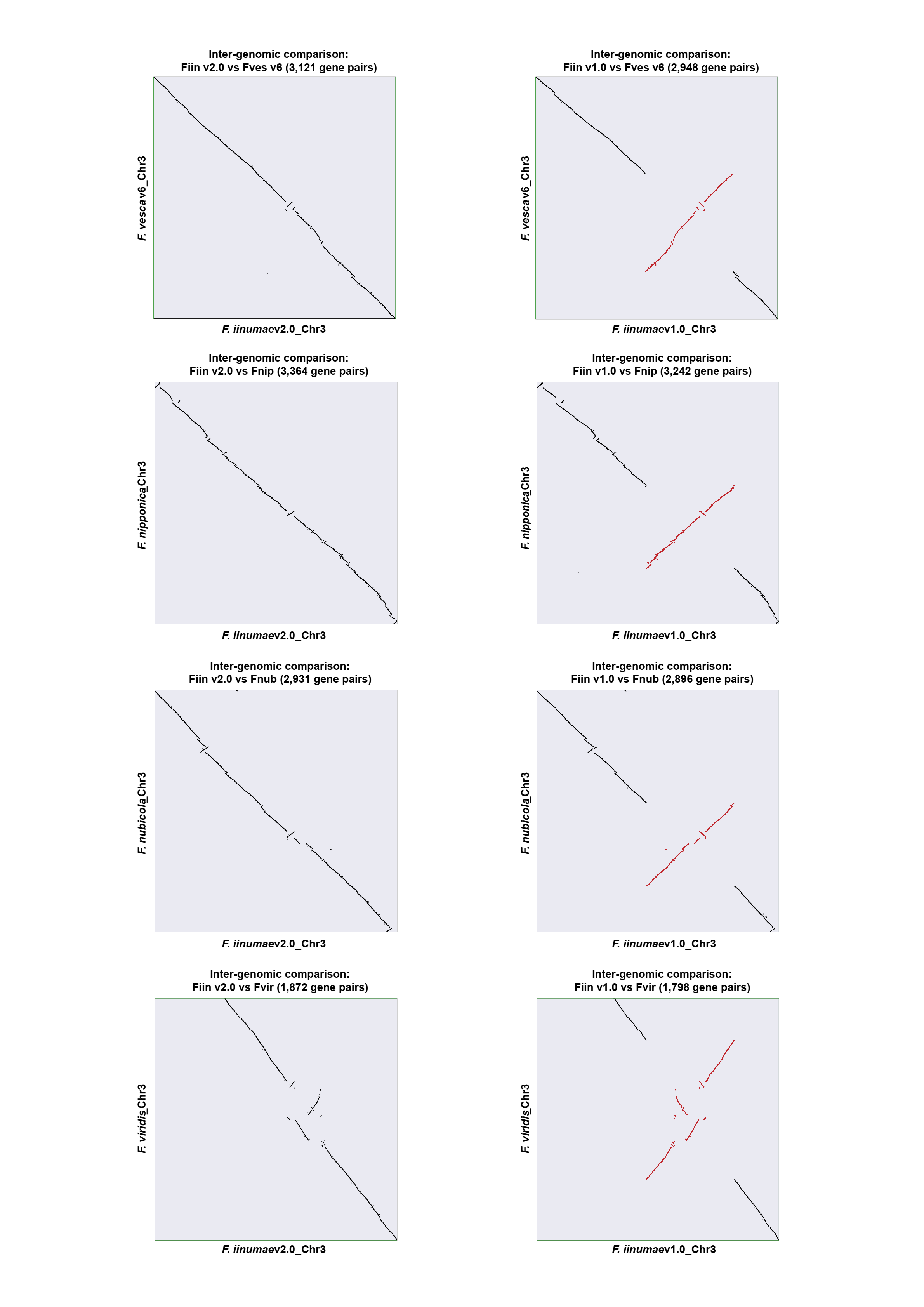


**Supplementary Figure 4 | Graphical alignment of *F. iinumae* chromosome 3 (v1.0 and v2.0) with the *F. vesca*, *F. nipponica*, *F. nbuicola* and *F. viridis* genomes.**


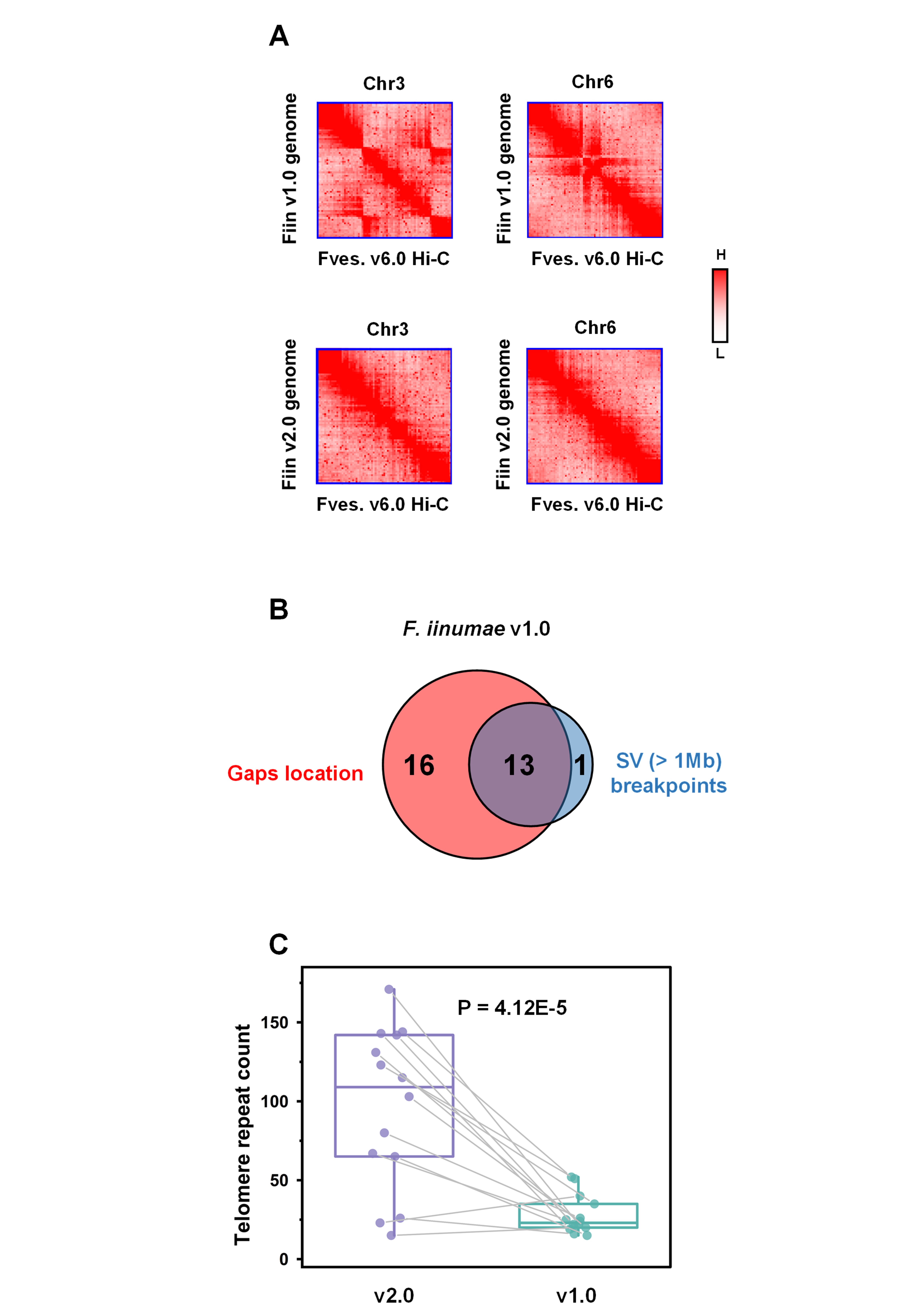


**Supplementary Figure 5 | Investigating putative structural variants and telomere repeat count between *F. iinumae* v1.0 and v2.0 assemblies. (A)** Mapping of Hi-C reads of *F. vesca* v 6.0 onto the two assemblies of *F. iinumae*. **(B)** Venn diagram illustrating the overlap between gaps in *F. iinumae* v1.0 and structural variant (SV) breakpoints. **(C)** Difference in telomere repeat count between *F. iinumae* v2.0 and v1.0 (independent samples t-test).


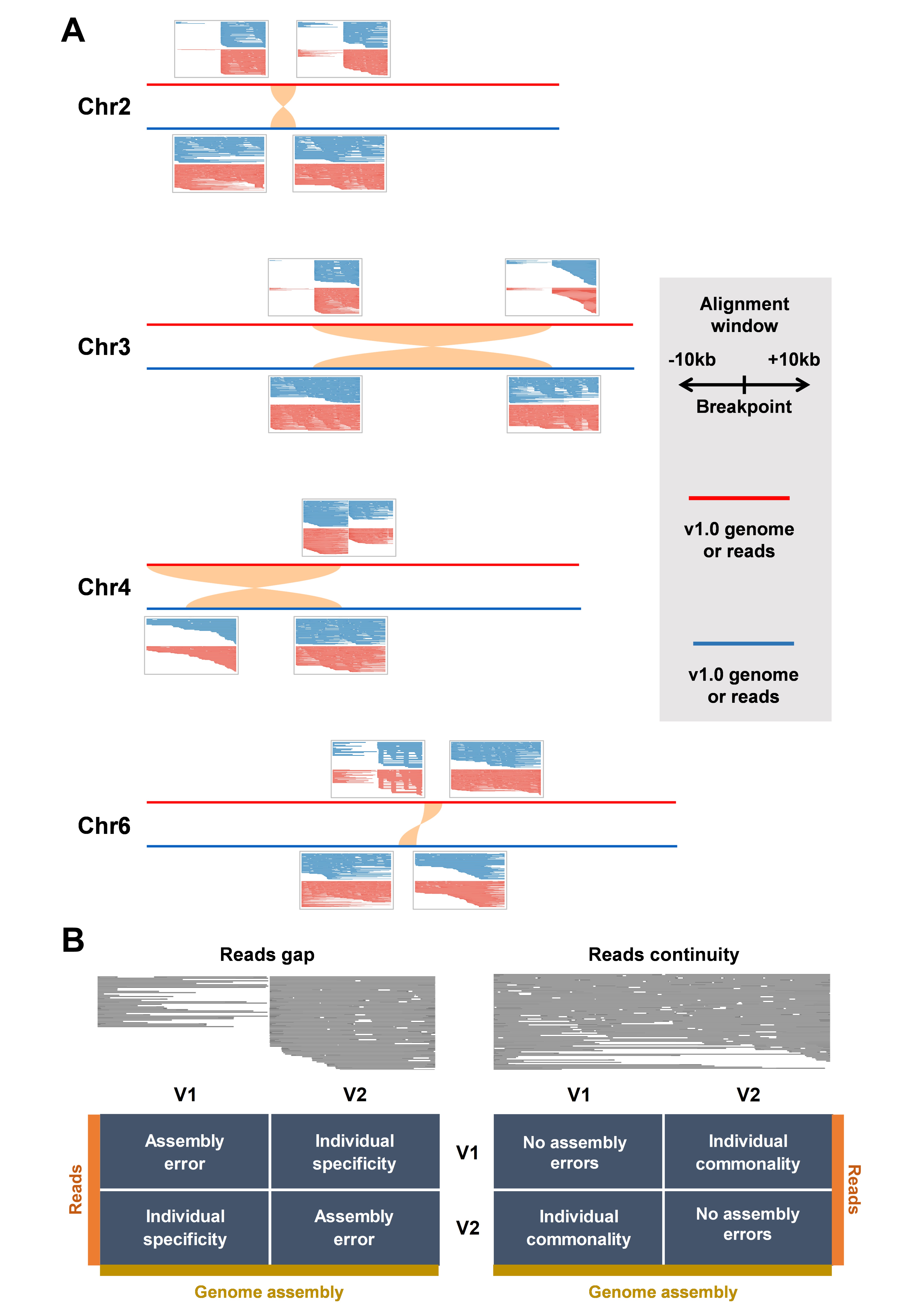


**Supplementary Figure 6 | Validation of structural variations. (A)** Validation of structural variations based on Pacbio reads mapping. **(B)** Summary of reasons for reads gap and continuity.


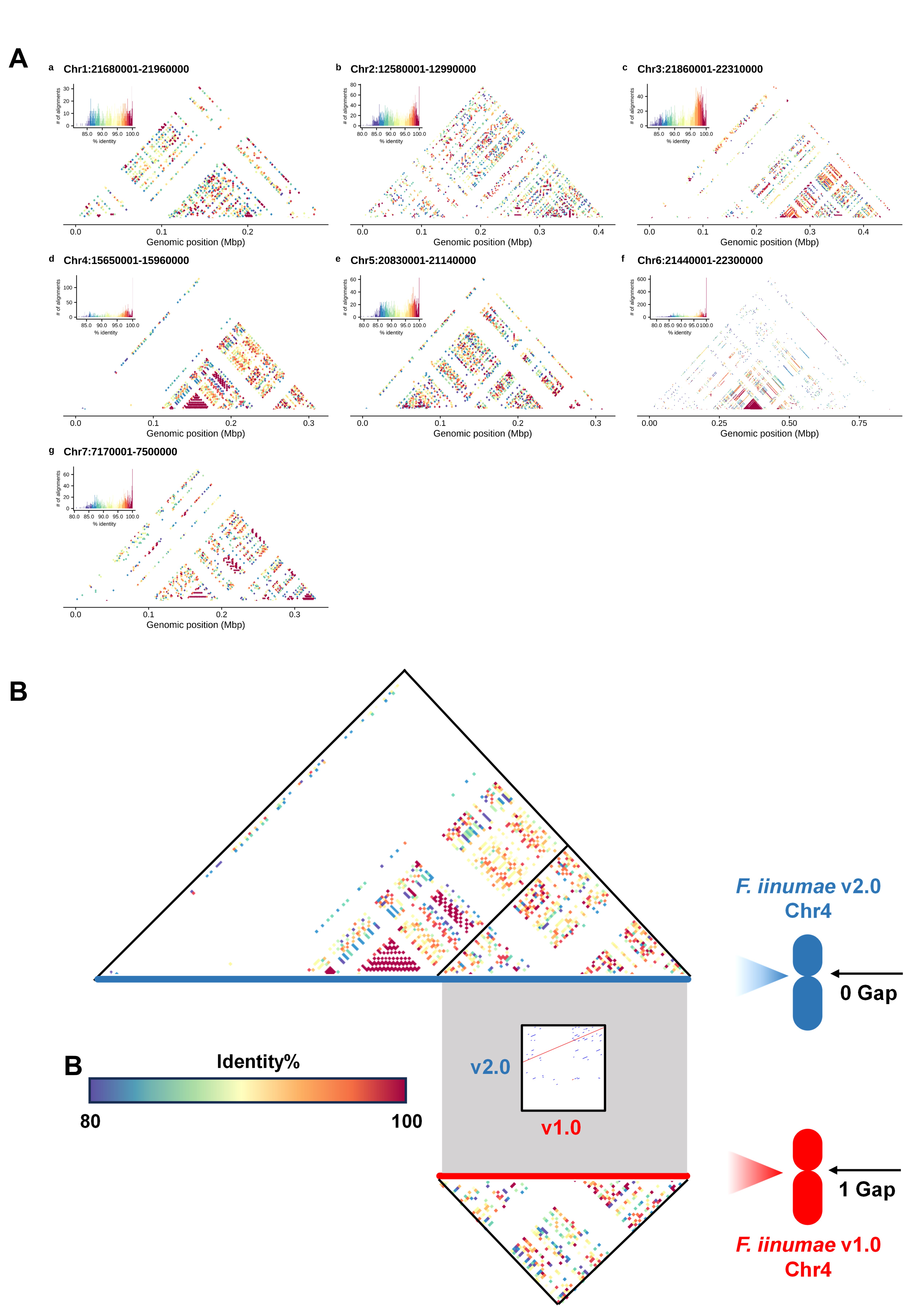


**Supplementary Figure 7 | Centromere structural features (A)** Centromere structural features of *F. iinumae* v2.0. **(B)** Comparison of centromere structure features between Chr4 of v1.0 and v2.0.


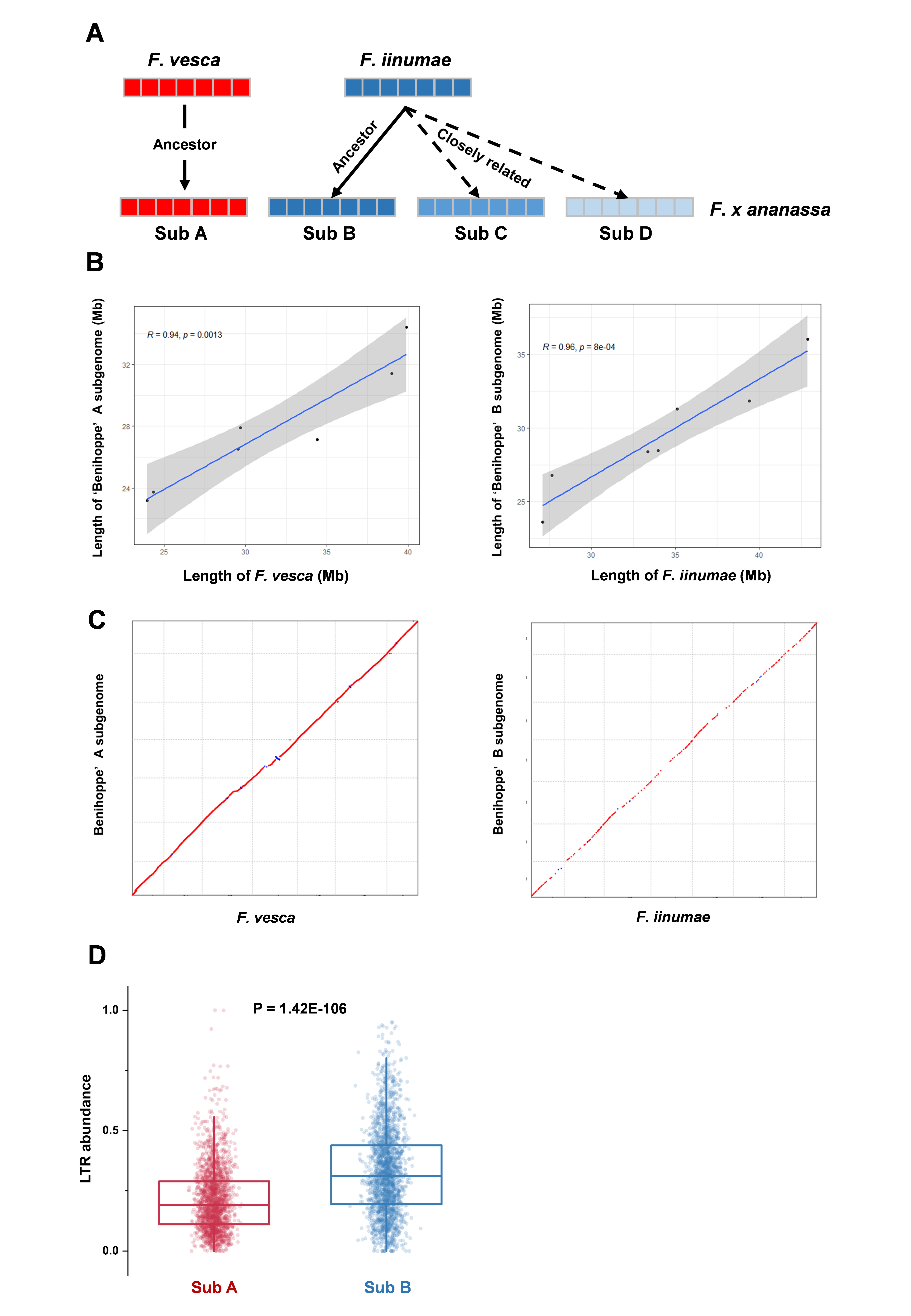


**Supplementary Figure 8 | Genome features of diploids and subgenomes in octoploid strawberries. (A)** Correspondence of the subgenome of octoploid strawberries with *F. vesca* and *F. iinumae*. **(B)** Correlation of chromosome lengths between *F. vesca* and subgenome A. Correlation of chromosome length between *F. iinumae* and subgenome B. **(C)** Genome-wide colinearity of *F. vesca* with subgenome A. Genome-wide colinearity of *F. iinumae* with subgenome B. **(D)** Difference in LTR abundance between ‘Benihoppe’ subgenome A and B (independent samples t-test).


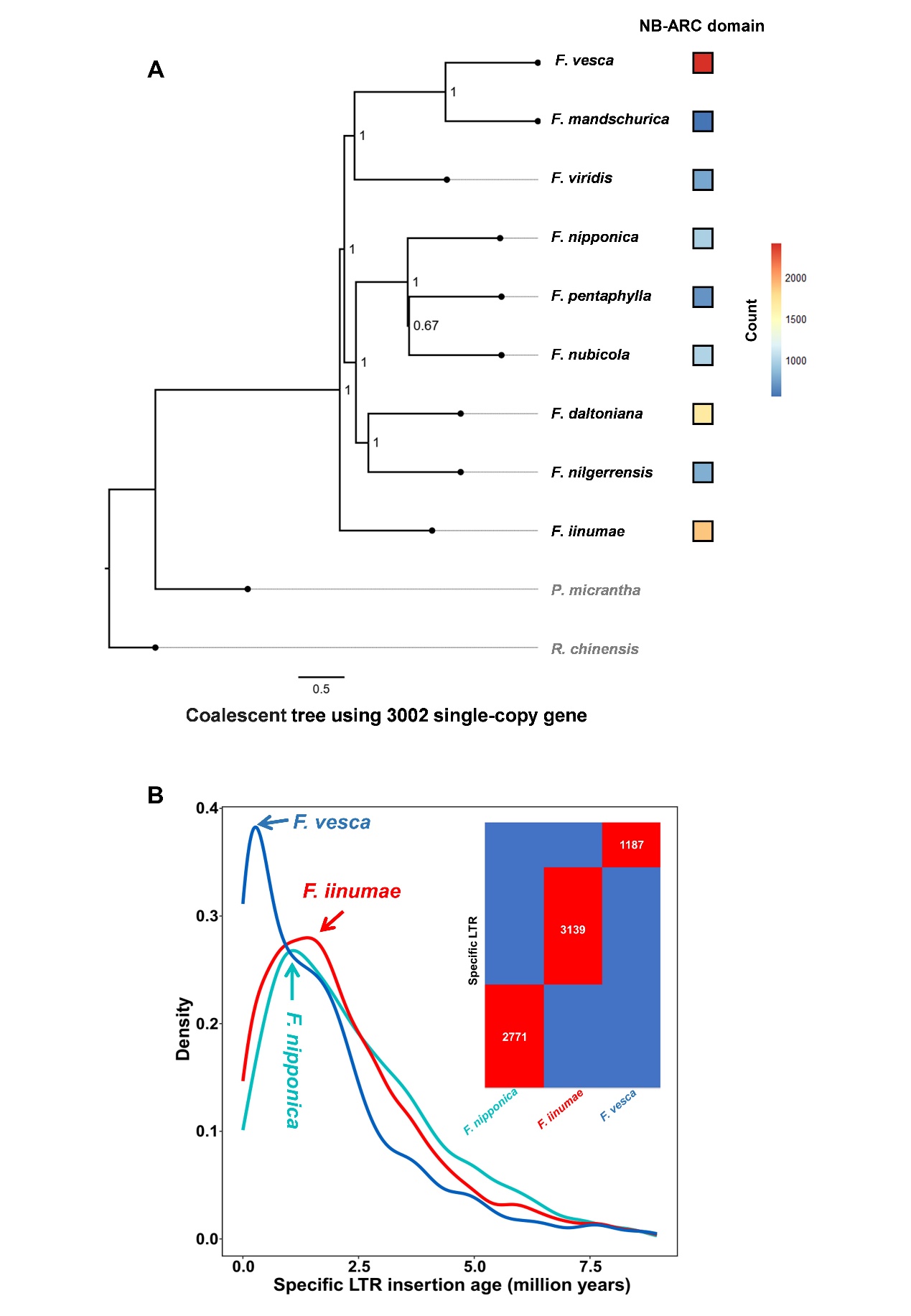


**Supplementary Figure 9 | Determination of the phylogenetic position of *Fragaria iinumae*. (A)** Coalescent-based analysis of 3002 genes from nine diploid *Fragaria* species (left) and distribution of NLRs across these species (right). **(B)** Estimation of the insertion time of LTRs in three representative diploid species.


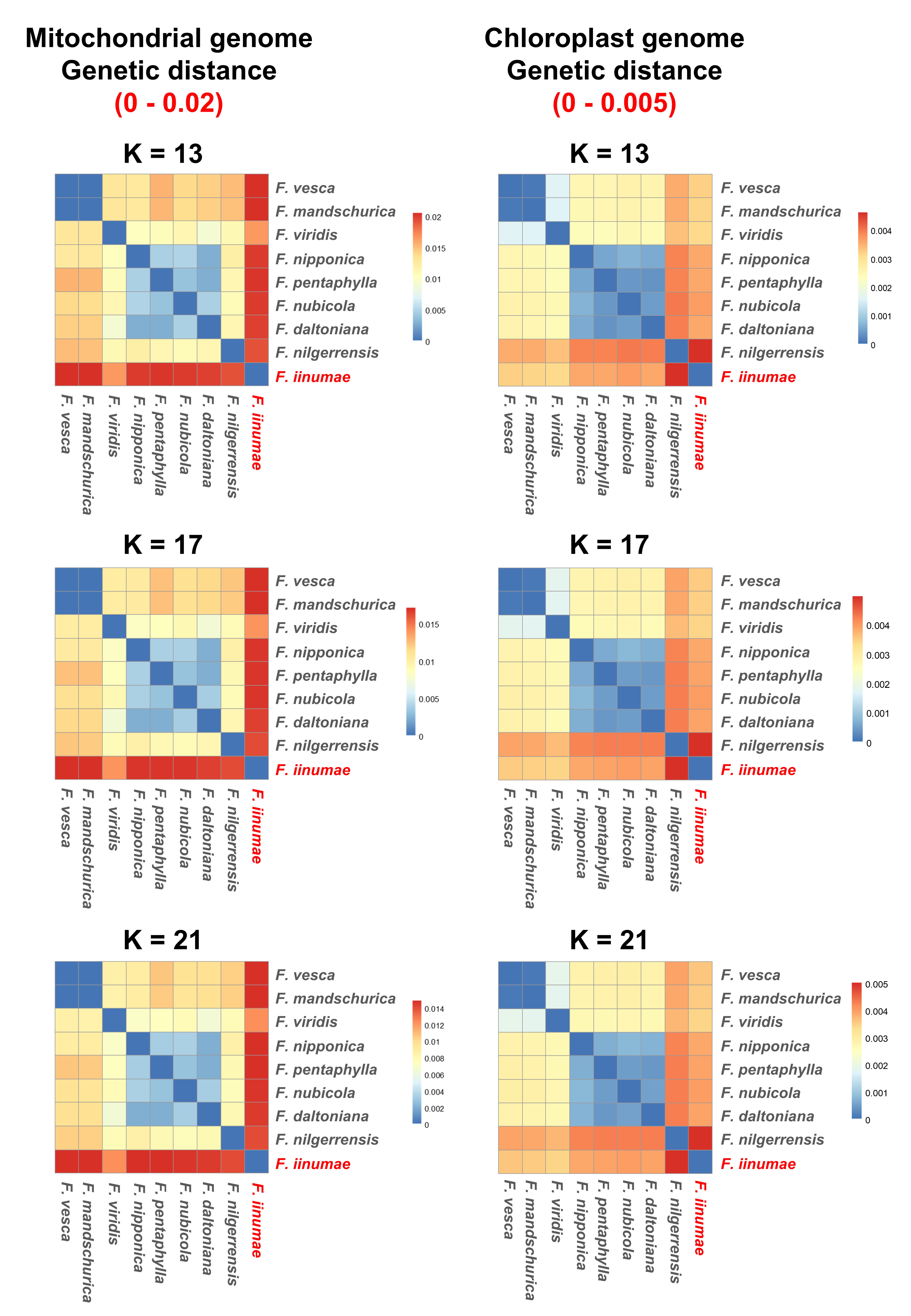


**Supplementary Figure 10 | Genetic distance matrix among nine diploid plastid genomes based on k-mer calculations with sizes of 13, 17, and 21.**

**
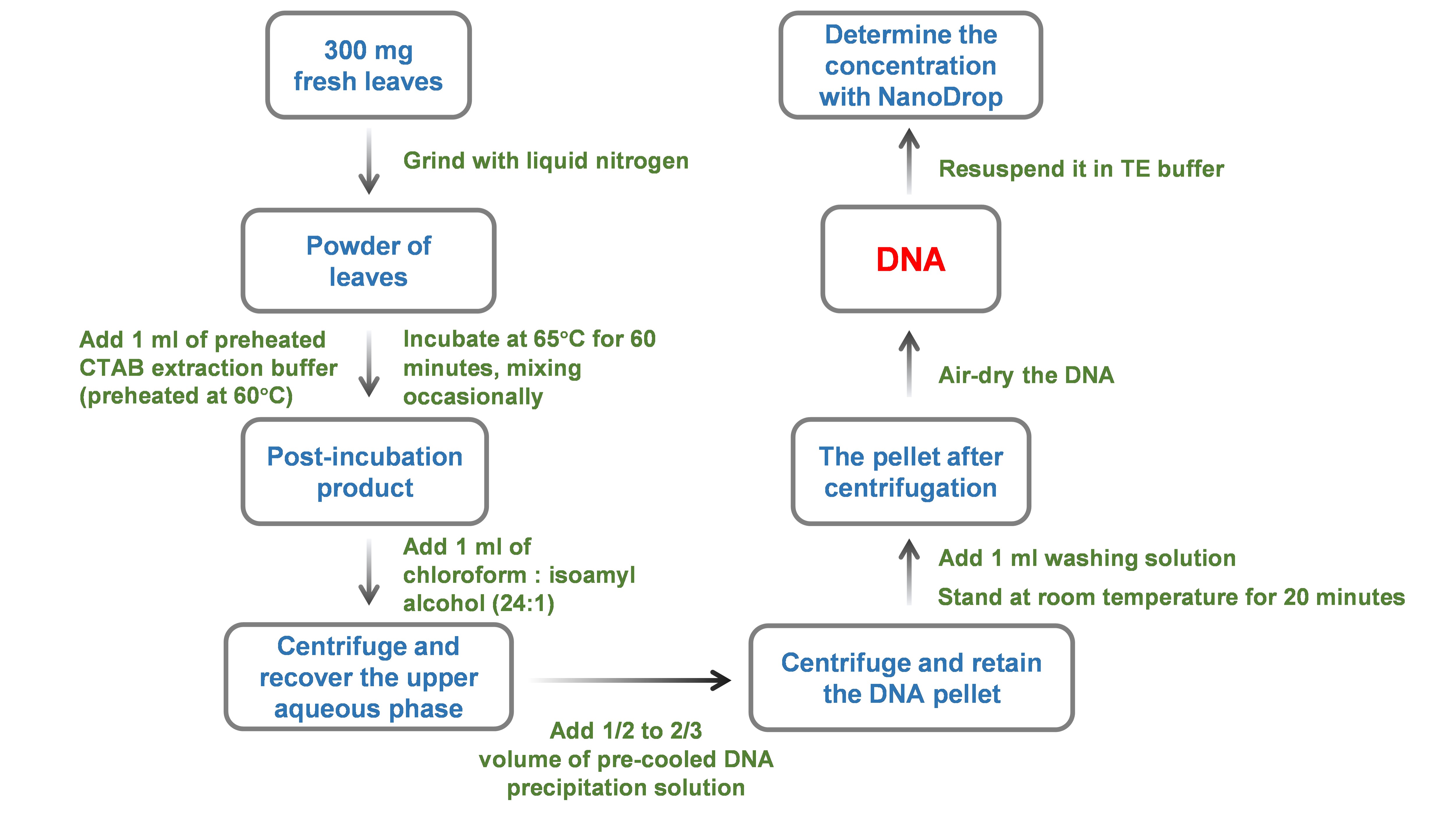
**

**Supplementary Figure 11 | Flowchart illustrating the CTAB protocol used for DNA extraction.**

**Supplementary tables**

**Table S1. Summary of genome feature and quality metrics for the *F.iinumae* v2.0 assembly**

| **Category** | **Fiin v1.0** | **Fiin v2.0** |
| --- | --- | --- |
| **Sequencing:** |  |  |
| PacBio (Gb) | 45.8 | 26.7 |
| Hi-C (Gb) | / | 33.4 |
| Genetic map | √ | / |
| **Genome estimate:** |  |  |
| Ploidy level (x = 7) | 2n = 2x = 14 | 2n = 2x = 14 |
| Genome size by k-mer analysis (Mb) | 265.6 | 242.6 |
| **Assembly feature:** |  |  |
| Assembled Genome Size (Mb) | 240.6 | 241.1 |
| Total number contigs | 94 | 17 |
| Length of contig N50 (Mb) | 10.7 | 33.3 |
| Assigned rate (%) | 99.4 | 99.4 |
| Number of gaps | 29 | 0 |
| Number of centromeres | 7 | 7 |
| Number of telomeres | 14 | 14 |
| LAI value | 16.2 | 16.3 |
| Merqury QV | 49.3 | 47.6 |
| Merqury completeness (%) | 98.9 | 99.0 |
| **Annotation feature:** |  |  |
| Repeat density (%) | 42.7 | 44.6 |
| Number of genes | 23665 | 27809 |
| BUSCO completeness (%) | 95.4 | 96.4 |
| OMArk completeness (%) | 96.1 | 96.2 |

**Table S2: Identification of repetitive sequences in *F. iinumae* v2.0 genome**

| **Total repeat fraction** | **Length (bp)** | **% of genome** |
| --- | --- | --- |
|  | **106747029** | **44.56%** |
| Class I: Retroelement | 73,457,524 | 30.66% |
| LTR Retrotransposon | 70,140,194 | 29.28% |
| Ty1/Copia | 15,407,955 | 6.43% |
| Ty3/Gypsy | 52,172,245 | 21.78% |
| Other | 135,980 | 0.06% |
| Non-LTR retrotransposon | 3,317,330 | 1.39% |
| LINE | 2,967,433 | 1.24% |
| SINE | 349,897 | 0.15% |
| Class II: DNA transposon | 15,115,815 | 6.31% |
| hobo-Activator | 2,884,746 | 1.20% |
| Tc1-IS630-Pogo | 131,986 | 0.06% |
| MULE-MuDR | 3,799,929 | 1.59% |
| PiggyBac | 171,472 | 0.07% |
| Tourist/Harbinger | 1,643,578 | 0.69% |
| Unclassified retroelement | 14,733,928 | 6.15% |

**Table S3. Location of telomeres and centromeres in the *F. iinumae* v 2.0 gneome**

|  | |  | **Telomeres** | | | | **Centromeres** | |
| --- | --- | --- | --- | --- | --- | --- | --- | --- |
| **Chr ID** | **Length** | | **Start** | **End** | **Start** | **End** | **Start** | **End (bp)** |
| Chr1 | 27,664,304 | | 1 | 1,268 | - | - | 21,680,000 | 21,960,000 |
| Chr2 | 33,360,077 | | 12 | 569 | 33,360,007 | 33,360,074 | 12,580,000 | 12,990,000 |
| Chr3 | 39,425,077 | | 7 | 1,198 | 39,424,055 | 39,425,077 | 21,860,000 | 22,310,000 |
| Chr4 | 35,125,333 | | 19 | 635 | 35,124,264 | 35,125,333 | 15,650,000 | 15,960,000 |
| Chr5 | 33,990,673 | | 2 | 477 | 33,990,203 | 33,990,673 | 20,830,000 | 21,140,000 |
| Chr6 | 42,903,465 | | 1 | 1,381 | 42,903,231 | 42,903,465 | 21,440,000 | 22,300,000 |
| Chr7 | 27,099,575 | | - | - | 27,098,231 | 27,099,575 | 7,170,000 | 7,500,000 |

**Table S4 | Sequence of centromeric and telomeric satellites**

| >CentromereRepeats (147 bp) ATTTTCGCACGATCTTACGAGATTGCGAACATATCAACGTTCTAGGAATCTGCCGAAA  CCAAATTCTTGGCTCTTTTTGGGACCTCCATACACCCACAACATTGATCTTTCAACTTT  TTTTCAGAAAATTTGACGTGTTATTTTGGC  >TelomereRepeats (7 bp)  TTTAGGG/CCCTAAA |
| --- |
